# Supplementary material for: Understanding Alternatives to Tobacco Production in Kenya: A Qualitative Analysis at the Sub-National Level
Source: Int J Environ Res Public Health. 2020 Mar 19;17(6):2033. doi: 10.3390/ijerph17062033 (PMC7143228; doi:10.3390/ijerph17062033)
Supplement: Supplementary file 1 [file ijerph-17-02033-s001.pdf]

Supplementary Table 1. Additional Quotes on Theme 1 (Tobacco).

Category	Perceived benefits			Stated Challenges			
Sub-Category	Predictable market	Perceived profitability	Sustainable land use	Monetary losses	Labor intensive	Health risk	Environmental degradation
Selected quotes	"Farmers knew the market, so they were growing knowing the market. At no one time it reached a place where you produce and tobacco was not taken from the field." – Agribusiness Officer<br><br>"Tobacco's value chain is organized because it is mainly driven by the processor. So as a crop, it is a crop which is being pulled by the market." – Sub-County Agricultural Officer	"[Tobacco] is a cash crop, so it is fetching farmers cash." – Agribusiness Officer<br><br>"You can sell the tobacco in bulk and get your money in lump sum." – Farmer<br><br>"Tobacco can help improve one's lifestyle, when you sell it you can build a plot which will help you in the future." – Farmer	"[British American Tobacco] were also providing tree seedlings, agroforestry tree seedlings. You find that in regions where tobacco was grown, there was an increased tree cover in the farm. That also attracted enhanced rainfall in those regions." – Agribusiness officer	"Once you reach the loan estimate they don't take the rest." – Farmer<br><br>"Some sold their tobacco in 2014 they haven't been paid to date" – Farmer<br><br>"Because grading is done by you and me I can easily downgrade your tobacco… I have gotten less money than what I was expecting." – Crops Officer<br><br>"When you get to quantify how much you've used vis-à-vis the margin that you've got, you realize that the margin that you	"Tobacco work goes for 24 hours the weeding, harvesting, drying, burning, you will even sleep outside." – Tobacco farmer<br><br>"The non-growers of tobacco are well because they have time to engage in other activities while tobacco farmers don't have time." – Farmer<br><br>"They [farmers] engage their children, the whole household members are busy doing something, from planting and preparation all	"Farmers who were struggling with cancer or struggling with asthma, chest problems, attributes of tobacco." – Sub-County Agricultural Officer<br><br>"A farmer just, when he is spraying, he or she wears their own clothes. I think the contact with the chemicals is too much." – Sub-County Agricultural Officer<br><br>"They [farmers] may be inhaling a lot of the tobacco while drying." – Sub-	"It's a heavy feeder, especially nitrogen… allelopathic chemicals that they exude in the soil. So all these things combine together and the soil becomes seriously depleted." – Sub-County Agricultural Officer<br><br>"We see the environment being destroyed. If you look around you can see there are very few trees. The reason is that this crop requires a lot of firewood when it comes to processing or drying." – Sub-County Agricultural Officer

			get is so little, often times it is negative." – Sub-County Agricultural Officer<br><br>"The cost of inputs is very high. They [companies] exaggerate the costs…" – Sub-County Agricultural Officer	the way to the market." – Sub-County Agricultural Officer	County Agricultural Officer	

Supplementary Table 2. Additional Quotes on Alternative Crops.

Category	Challenges			Opportunities	
Sub-Category	Lack of Market	Low Prices	Pests and Diseases	High Returns	Low Barriers of Production
Cereals	"If maize had a bulk market and with good prices I would have supported maize growing." – Tobacco farmer<br><br>"Maize is a product that everyone is doing in Kenya… it is a market that has been diluted" – Sub-County Agricultural Officer	"It's a food security crop for them, they will not mind using 20,000KSH to get 6 or 7 bags of maize in an acre… they haven't even made a profit. But they'll still insist on growing maize." – Agribusiness Officer	"Currently we are suffering, even for maize crops pests have invaded and the harvests are low" – Farmer<br><br>"Now we have fall armyworm attacking our maize" – Sub-County Agricultural Officer	"Sorghum has a good market… no fluctuating prices, sorghum may end up helping us more than tobacco… When you do comparison of the work input required for each crop, sorghum brings in more money" – Tobacco farmer<br><br>"Maize we will get two seasons of production. So there are even more returns than compared to tobacco" – Sub-	"You prepare the farm and wait for the rains to plant the maize seeds, they require only two weeding sessions then you wait for the harvest time. In between the weeding sessions, you apply fertilizer or you can apply it during planting time. Harvesting takes just one day" – Farmers

				County Agricultural Officer	
Tubers	"We are focusing much on cassava because we have other stakeholders who are coming in to assist us in marketing. The challenge that we have is not production, the challenge is on marketing in terms of pricing, where they are selling" – Sub-County Agricultural Officer	"Potato farming is good but the prices are not constant. Prices can drop from 1700 to 1000 or 700 shillings per bag." – Farmer	"Cassava was almost wiped out of the county by two diseases: cassava mosaic virus and cassava brown streak disease" – Agribusiness Officer	" Nowadays; potatoes are our cash crop" – Farmer	"It is a resilient crop… So the cost of production is very low with sweet potato." – Sub-County Agricultural Officer
Vegetables	"if you grow a lot of tomatoes there is no market to sell them that's why brokers come and take two bags and buy them as one." – Farmer	" When you grow things like maize or beans you will never get cash" – Farmer		"Tomatoes farming will help me pay my children' school fees… they are profitable." – Farmer	
Horticulture				Horticulture is coming up; it is a high value crop. It has got returns [more] than the other… these crops are emerging because of their value" – Sub-County Agricultural Officer	"[horticulture crops] are friendly, they can grow naturally, though some we have domesticated, so the conditions are favorable for them to grow" – Sub-County Agricultural Officer

Supplementary Guide 1: Interview Guides

Interview Guide: Government Officials

1. Can you please tell us about your position?
    a. How long have you been in this position?
    b. What are the main responsibilities?
    c. What would you say is your main mandate?
2. Agricultural development
    a. What are the key policies or plans that guide your work?
    b. What would you say are the main development targets for agriculture in your region?
        i. How have you been pursuing these targets?
        ii. Who have you been collaborating with?
    c. How were these targets established?
    d. How have these targets changed over the years?
    e. What crops do you think have been the most successful in terms of farmer livelihoods, farmer profits, etc.?
        i. Can you describe the supply chain for these crops?
            - How do they get to market?
            - Who purchases the crop?
            - Is the crop exported or does it stay within Kenya? Is the crop processed or manufactured in the region or sent away as a raw product?
3. Can you please tell us about tobacco growing in your district?
    a. What is your perspective on the tobacco supply chain?
    b. What is your perspective on tobacco as a viable crop for farmers?
        i. Can you share some stories from tobacco farmers or your own observations of tobacco growing?
    c. From your perspectives, what is the role of the leaf buying companies?
    d. What are some of the challenges or benefits of tobacco growing in your county?
4. Which other ministries or agencies do you work most closely with?
    a. What sort of work do you do together?
    b. Can you please describe your relationship with the health ministry?
        i. Have you had any contact with the health ministry around tobacco control?
5. Can you share your perspective on the future of agriculture in your region? (Is it promising? If so, why? If not, why?)
6. Is there anything we haven't asked that you would like to share about tobacco growing or economic development more generally in your county?

Interview Guide: Extension Workers

1. Can you describe your main responsibilities as an extension worker?
2. What does a typical day involve for you?
3. Do you work with farmers growing tobacco? If so, what is your involvement? If not, why?
    a. Can you explain how tobacco production (from preparation to growing to market) works in the region?
4. From your perspective what are some of the best crops being grown in your area? What makes them good?
5. To what extent does government policy guide agricultural production in your region?
6. What do you think the future holds for agriculture in your region?
